# The association between individual counselling and health behaviour change: the See Kidney Disease (SeeKD) targeted screening programme for chronic kidney disease

**DOI:** 10.1186/s40697-016-0127-4

**Published:** 2016-07-20

**Authors:** Lauren Galbraith, Brenda Hemmelgarn, Braden Manns, Susan Samuel, Joanne Kappel, Nadine Valk, Paul Ronksley

**Affiliations:** Department of Community Health Sciences, Cumming School of Medicine, University of Calgary, HSC G239, 3330 Hospital Drive NW, Calgary, Alberta T2N 4N1 Canada; Department of Medicine, University of Calgary, Calgary, Canada; Division of Nephrology, University of Saskatchewan, Saskatoon, Canada; Kidney Foundation of Canada, Ottawa, Canada

**Keywords:** Chronic kidney disease, Behaviour change intervention, Counselling

## Abstract

**Background:**

Health behaviour change is an important component of management for patients with chronic kidney disease (CKD); however, the optimal method to promote health behaviour change for self-management of CKD is unknown. The See Kidney Disease (SeeKD) targeted screening programme screened Canadians at risk for CKD and promoted health behaviour change through individual counselling and goal setting.

**Objectives:**

The objectives of this study are to determine the effectiveness of individual counselling sessions for eliciting behaviour change and to describe participant characteristics associated with behaviour change.

**Design:**

This is a cross-sectional, descriptive study.

**Setting:**

The study setting is the National SeeKD targeted screening programme.

**Patients:**

The participants are all ‘at risk’ patients who were screened for CKD and returned a follow-up health behaviour survey (*n* = 1129).

**Measurements:**

Health behaviour change was defined as a self-reported change in lifestyle, including dietary changes or medication adherence.

**Methods:**

An individual counselling session was provided to participants by allied healthcare professionals to promote health behaviour change. A survey was mailed to all participants at risk of CKD within 2–4 weeks following the screening event to determine if behaviour changes had been initiated. Descriptive statistics were used to describe respondent characteristics and self-reported behaviour change following screening events. Results were stratified by estimated glomerular filtration rate (eGFR) (< 60 and ≥ 60 mL/min/1.73 m^2^). Log binomial regression analysis was used to determine the predictors of behaviour change.

**Results:**

Of the 1129 respondents, the majority (89.8 %) reported making a health behaviour change after the screening event. Respondents who were overweight (body mass index [BMI] 25–29.9 kg/m^2^) or obese (BMI ≥ 30.0 kg/m^2^) were more likely to report a behaviour change (prevalence rate ratio (PRR) 0.66, 95 % confidence interval (CI) 0.44–0.99 and PRR 0.49, 95 % CI 0.30–0.80, respectively). Further, participants with a prior intent to change their behaviour were more likely to make a behaviour change (PRR 0.58, 95 % CI 0.35–0.96). Results did not vary by eGFR category.

**Limitations:**

We are unable to determine the effectiveness of the behaviour change intervention given the lack of a control group. Potential response bias and social desirability bias must also be considered when interpreting the study findings.

**Conclusions:**

Individual counselling and goal setting provided at screening events may stimulate behaviour change amongst individuals at risk for CKD. However, further research is required to determine if this behaviour change is sustained and the impact on CKD progression and outcomes.

## Abrégé

**Mise en contexte:**

Les changements dans les habitudes de vie sont une composante majeure dans la prise en charge des patients atteints d’insuffisance rénale chronique (IRC). Malgré cela, la méthode pour promouvoir efficacement ces changements auprès de cette clientèle particulière n’est pas connue. Le programme de dépistage précoce et ciblé *See Kidney Disease (SeeKD)* a permis d’identifier les Canadiens à risque de développer une IRC. Ce programme a aussi servi à promouvoir l’adoption de changements d’habitudes bénéfiques pour la santé, par le biais de consultations individuelles et par l’établissement d’objectifs.

**Objectifs de l’étude:**

Cette étude avait pour objectif de mesurer l’efficacité de séances de consultation individuelle offertes aux patients en vue de susciter des changements comportementaux. L’étude visait également à établir les caractéristiques des patients associées à ces changements de comportement.

**Cadre de l’étude:**

Il s’agit d’une étude transversale descriptive qui s’est tenue dans le cadre du programme national de dépistage ciblé *SeeKD*.

**Participants:**

La cohorte était constituée de tous les patients identifiés « à risque de développer une IRC » par le programme *SeeKD,* et ayant retourné le questionnaire de suivi au sujet des changements dans leurs habitudes de vie, soit un total de 1129 participants.

**Mesures:**

Une séance de consultation individuelle ayant pour but de promouvoir l’intégration de nouvelles d’habitudes de vie a été offerte aux participants par les professionnels de la santé à la suite de l’activité de dépistage. Entre deux et quatre semaines plus tard, les participants ont également reçu un questionnaire par la poste à l’aide duquel on a pu vérifier s’ils avaient entamé les changements de comportement proposés. Des statistiques descriptives ont été utilisées pour établir les caractéristiques des répondants ainsi que les changements de comportement que ces derniers ont rapporté avoir adoptés à la suite de l’activité de dépistage. Les résultats ont été stratifiés en deux groupes selon les valeurs de DFGe des participants (< 60 mL/min/1.73 m^2^ et ≥ 60 mL/min/1.73 m^2^). L’analyse par régression logistique binomiale a été utilisée pour identifier les indicateurs de changement de comportement chez les patients.

**Résultats:**

La grande majorité (89,8 %) des 1129 participants ont rapporté avoir adopté de nouveaux comportements en matière de santé après avoir été déclarés à risque de développer une IRC. Les répondants souffrant d’embonpoint (indice de masse corporelle [IMC] entre 25 et 29,9 kg/m^2^) ou obèses (IMC ≥ 30,0 kg/m^2^) se sont avérés plus ouverts à l’idée d’adopter de nouveaux comportements en regard de leur santé (rapport du taux de prévalence [RTP] : 0,66 ; intervalle de confiance à 95 % [I.C. à 95 %] : entre 0,44 et 0,99 et RTP : 0,49 ; I.C. à 95 % : entre 0,30 et 0,80 respectivement). Par ailleurs, les répondants qui avaient déjà l’intention d’adopter de nouveaux comportements avant même d’être dépistés ont été les plus enclins à le faire (RTP : 0,58 ; I.C. à 95 % : entre 0,35 et 0,96). Aucune variation significative de ces résultats n’a été observée selon le DFGe.

**Limites de l’étude:**

Nous n’avons pu déterminer avec précision l’efficacité des changements de comportement adoptés en raison de l’absence d’un groupe contrôle. De plus, un biais dû aux réponses des participants ou par désirabilité sociale est à considérer dans l’interprétation des résultats.

**Conclusions:**

L’établissement d’objectifs ainsi que le counselling individuel fourni à la suite de l’activité de dépistage pourraient stimuler l’adoption de nouvelles habitudes de vie chez les patients à risque de développer une IRC. Toutefois, des recherches supplémentaires sont requises afin de déterminer si ces changements de comportement sont maintenus par les patients et s’ils ont une réelle influence sur le pronostic de la maladie.

## What was known before

Health behaviour change is an important aspect for the management of patients with chronic kidney disease (CKD).

## What this adds

Individual counselling and goal setting provided at the screening events may stimulate behaviour change amongst individuals at risk for CKD. However, participants who were identified as having lower eGFR (< 60 mL/min/1.73 m^2^) were not more likely to change their behaviour given their recent diagnosis at the screening event. Further research is required to determine if this behaviour change is sustained and the impact on CKD progression and outcomes.

## Background

CKD is associated with an increased risk for cardiovascular disease and concomitant chronic illness [1, 2]. Progression to end-stage renal disease (ESRD) has traditionally been considered the most serious complication of CKD [3] given its association with high morbidity and mortality [4, 5]. However, the majority of patients with CKD die prematurely from CKD-related complications before progressing to ESRD [6, 7]. Consequently, compliance with chronic disease management such as blood pressure control [8, 9], glycaemic control [10–12], and use of statins [13] is critical to slowing the progression to ESRD, preventing vascular-related adverse outcomes and reducing the risk of premature mortality [14]. In addition to the use of medications, management of chronic medical conditions including CKD requires lifestyle (behaviour) changes. This relates to the transformation or modification of behaviours by addressing knowledge, attitudes, and practices. Promoting behaviour change, through improving patient motivation and health knowledge, has been identified as a key component of chronic disease management given the known association between poor health behaviours and adverse clinical outcomes [15, 16].

Michie et al. [17] identified three core components to behaviour change: capability, motivation, and opportunity. While educational interventions build capability for behaviour change [18–20], research suggests that healthcare professionals play an important role in providing motivation and opportunity for behaviour change [21]. Specifically, individual counselling has been identified as a potentially effective intervention to improve health behaviour change within various chronic conditions (diabetes and hypertension) [22, 23]. Although evidence is limited in CKD, behaviour change interventions have focused on overall health and lifestyle changes (namely diet modification). Though not specific to CKD management, these interventions which aim to improve overall quality of life and slow kidney progression have shown promise in reducing CKD-related symptoms and complications [24]. However, given the heterogeneous interventions published and a paucity of evidence, the optimal method to elicit behaviour change within the CKD population remains unknown [25].

The Kidney Foundation of Canada launched the See Kidney Disease (SeeKD) targeted screening programme for Canadians at risk of CKD to promote early detection of CKD and to improve health knowledge in CKD management through individual counselling and goal setting provided at the screening events. We sought to determine the effectiveness of the individual counselling sessions for eliciting behaviour change amongst participants and to describe the participant characteristics associated with self-reported behaviour change.

## Methods

The Kidney Foundation of Canada conducted the SeeKD targeted screening programme from 2011 to 2014 and recruited 6329 individuals across nine Canadian provinces, of whom 5194 were determined to be ‘at risk’ and subsequently screened for CKD. Eligible participants were adults 18 years of age and older who provided informed consent. Details of the SeeKD programme and methodology have previously been reported [26]. In brief, all participants that attended a screening event were surveyed to gather baseline sociodemographic characteristics, risk factors for CKD, knowledge of kidney disease, current health behaviours, and to determine those at risk of CKD. *At risk of CKD* was defined as having at least one of the following self-reported risk factors: diagnosed diabetes, diagnosed high blood pressure, existing kidney problems, family history of kidney disease, member of a high-risk ethnic population, current vascular disease, and currently using tobacco products. Only participants determined to be at risk of CKD were screened using point-of-care creatinine measurements (to calculate an estimated glomerular filtration rate (eGFR) and determine those with eGFR < 60 mL/min/1.73 m^2^). Participants were informed about the results, and if necessary, and with participant consent, results were forwarded to their family physician to arrange additional testing and follow-up. All surveys and educational documents were translated into the participant’s language of preference by the Kidney Foundation of Canada.

Immediately following kidney-specific testing, an individual counselling and goal setting session was provided to each participant determined to be at risk of CKD, with the goal of promoting health behaviour change amongst participants. Each one-on-one counselling session lasted approximately 20 min and was delivered by a registered nurse, pharmacist, or dietician specializing in kidney disease (Appendix 1). These counselling sessions provided educational information about CKD and its management and provided the participants with specific strategies tailored to their needs based on clinical measures taken during the screening and participants’ responses to the pre-screening survey. In the pre-screening survey, participants answered questions about their health knowledge of CKD (e.g. ‘Which of the following are risk factors for kidney disease?’), their motivation to participate in the screening event (e.g. ‘What made you participate in the SeeKD screening event today?’), and intent to change their health behaviours (e.g. ‘Are you planning to make any changes to improve your health?’ and ‘If you could change a health behaviour which one or two would be most important?’). We categorized intent to change behaviour as no intent to change any health behaviours, an expressed intent to change health behaviours, and preliminary health behaviour changes recently started.

Approximately 2–4 weeks after the SeeKD screening events, a follow-up survey was mailed to participants who received the individual counselling session and provided consent to be contacted. The post-screening survey sought to determine whether participants had begun to make health behaviour changes as recommended through the individual counselling sessions. The primary outcome of ‘health behaviour change’ was defined as a self-reported positive response to the post-screening question ‘Have you made any changes to improve your health in the past two weeks?’ Participants could choose more than one response from a predetermined list of health behaviour changes which were broadly categorized into common themes including the following: dietary changes (e.g. reducing fat or salt intake or adhering to Canada’s food guide); improving adherence to recommendations and prescriptions from healthcare providers (e.g. taking medications as prescribed, monitoring blood pressure or sugars, or routine visits to physician); reducing health-risk behaviours (e.g. quitting smoking or reducing alcohol intake); and daily lifestyle changes (increasing daily activity, reducing stress, or weight loss). Responses from participants who chose ‘other’ and indicated a specific health behaviour change were manually coded into the binary behaviour change variable during data cleaning. The response of ‘no health behaviour change’ was determined if the participant did not choose any of the suggested behaviour changes on the predetermined list or if they selected other and indicated they had not made any behaviour changes following the SeeKD screening event.

### Analysis

Descriptive statistics were used to characterize participants that responded to both the pre- and post-screening surveys. These characteristics include sociodemographic (age, sex), clinical characteristics (eGFR, BMI), self-reported risk factors for CKD, self-reported motivation to participate in screening, health knowledge of risk factors for CKD, and self-reported behaviour change. Specifically, age was categorized as ≤ 49, 50–64, and ≥ 65 years and BMI was categorized as ≤ 24.9, 25–29.9, and ≥ 30 kg/m^2^. Motivation to participate was reported in four groups (no specified motivation, concerned for personal health status, influenced by external sources, and recruitment efforts) while self-reported health knowledge and behaviour change was reported as dichotomous (yes/no) variables. Participant characteristics were also compared amongst those that did and did not respond to the post-survey to determine whether these groups differed systematically. Descriptive statistics were reported using numbers and proportions for categorical variables and means with standard deviations (SD) for normally distributed continuous variables.

We fit multivariable log binomial regression models to determine the prevalence rate ratios (PRRs) for characteristics associated with the primary outcome of health behaviour change. Selection of characteristics to include within our regression models was a balance between factors previously associated with health behaviour change amongst chronic disease populations [18–25] and those available within the patient survey. Given that the prevalence of self-reported health behaviour change was very high (89.8 %), we modeled the outcome of *no behaviour change*. The interpretation of a negative outcome (no behaviour change) is difficult. For example, a PRR of < 1.0 translates to a participant being less likely to make no behaviour change (alternatively stated, more likely to make a behaviour change). Consequently, we interpret the PRR and 95 % confidence intervals (CIs) in terms of a positive self-reported health behaviour change in our Results and Discussion sections.

We constructed models and tested variables for inclusion (using *p* < 0.05) that had been identified a priori as being potentially associated with the outcome. These candidate predictors of behaviour change were considered on the basis of previous literature and clinical relevance. Variables that were independent predictors of behaviour change through bivariate analysis, along with age and sex, were then used to create a full model. Backward elimination was used to create the most parsimonious model. Model fit was assessed using the Bayesian information criterion (BIC) where the model with the lowest BIC is preferred.

Regression analysis using eGFR category (< 60 or ≥60 mL/min/1.73 m^2^) as a potential effect modifier was attempted, but the model did not converge due to a small sample size. Consequently, a stratified analysis was conducted to determine if characteristics related to health behaviour change varied by eGFR category. Specifically, we hypothesized that participants who were identified as having lower eGFR (< 60 mL/min/1.73 m^2^) may be more likely to change their behaviour given their recent diagnosis at the screening event. Variables independently associated with the outcome of health behaviour change, determined through log binomial regression, were stratified by eGFR category. Results were compared using Pearson’s chi-square tests for proportions, the Wilcoxon rank-sum test for multi-level categorical variables, and *t* tests for continuous variables. Finally, we conducted a sensitivity analysis excluding participants who self-reported having kidney problems amongst those with an eGFR of < 60 mL/min/1.73 m^2^ to determine the potential influence on participant characteristics and whether those with a new diagnosis of eGFR < 60 mL/min/1.73 m^2^ were more motivated to change their behaviour than those with more longstanding kidney disease. No imputation methods were used to account for the small proportion of patients with missing data (*n* = 7). Rather, footnotes were included below all descriptive analyses where denominators were influenced by missing data. Further, all patients with missing data were excluded from the regression models.

The SeeKD targeted screening programme obtained research ethics board approval from Health Canada. Ethics approval for analysis was also obtained from the Conjoint Health Research Ethics Board at the University of Calgary. All statistical analyses were conducted using Stata, version 12 [27].

## Results

**Fig. 1:**
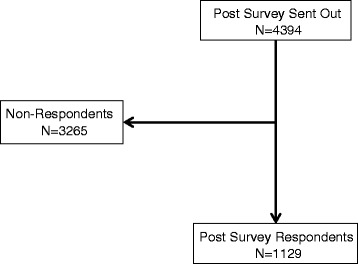
Participant flow chart

Overall, 5194 participants of the SeeKD programme were screened for CKD, of whom the majority (84.6 %) consented to receiving a post-screening follow-up survey, and 26 % responded (Fig. 1). The majority of the 1129 participants who responded were females (70.1 %) with a mean age of 63.8 years and were overweight or obese (33.3 and 27.9 %, respectively) (Table 1). Approximately, one in five (20.6 %) respondents had an eGFR of < 60 mL/min/1.73 m^2^ and the most common self-reported risk factors for CKD were hypertension (45.5 %) and member of a high-risk ethnic population (45.1 %). The majority of respondents were aware of at least one risk factor for CKD (health knowledge, 90.1 %), and their predominant motivation for participating in the screening events was a personal concern for health status (54.7 %).

**Table 1:** Participant characteristics amongst respondents to post-screening survey

	Respondents (*N* = 1129^a^)
Gender, male, *n* (%)	337 (29.9)
Age (years), mean (SD)	63.8 (14.3)
Age (years), *n* (%)	
≤ 49	183 (16.3)
50–64	345 (30.8)
≥ 65	594 (52.9)
eGFR < 60 mL/min/1.73 m^2^, *n* (%)	208 (20.6)
Self-reported behaviour change, *n* (%)	1014 (89.8)
Motivation for participating, *n* (%)	
Concern for personal health status	618 (54.7)
Influence from external source	227 (20.1)
Recruitment efforts	361 (32.0)
None	110 (9.7)
Self-reported risk factors, *n* (%)	
Diagnosed diabetes	274 (24.3)
Diagnosed hypertension	524 (45.5)
Problems with kidneys	156 (13.8)
High-risk ethnic groups	509 (45.1)
Vascular disease	268 (23.7)
Family history of kidney problems	166 (14.7)
Smoking or tobacco use	128 (11.3)
Knowledge of risk factors for CKD, *n* (%)	
Yes	1017 (90.1)
No	110 (9.7)
Body mass index, *n* (%)	
Normal/underweight (≤ 24.9 kg/m^2^)	385 (34.1)
Overweight (25.0–29.9 kg/m^2^)	376 (33.3)
Obese (≥ 30.0 kg/m^2^)	315 (27.9)

*SD* standard deviation, *CKD* chronic kidney disease, *eGFR* estimated glomerular filtration rate

^a^Denominators vary for each variable depending on the number of participants with complete data available

When comparing individuals who responded to the post-screening survey to those who did not, we found that a higher proportion of respondents were females (70.1 vs. 65.6 %), older (mean age 63.8 years vs. 56.5 years), and had a BMI in the normal or underweight category (BMI ≤ 24.9) (34.1 vs. 31.1 %) (Appendix 2). Further, more respondents self-reported hypertension (45.5 vs. 36.3 %, respectively), although non-respondents were more likely to be members of high-risk ethnic groups. Finally, respondents were more likely to be aware of the risk factors for CKD (health knowledge) than non-respondents (90.1 vs. 87.7 %, respectively).

The majority (89.8 %) of participants self-reported a health behaviour change in the post-screening survey. Amongst those who reported making a health behaviour change, most people indicated making dietary changes (79.9 %), improving their adherence to recommendations provided by their healthcare providers (65.7 %), and making daily lifestyle changes (75.8 %). A small proportion (6.4 %) of respondents indicated quitting smoking, chewing tobacco, or reducing alcohol intake as their health behaviour change (Table 2).

**Table 2:** Proportion of respondents who self-reported a behaviour change, by category of change

Categories of behaviour change	Respondents^a^ (*N* = 1014)
Dietary, *n* (%)	810 (79.9)
Improving adherence, *n* (%)	666 (65.7)
Reducing risk behaviours, *n* (%)	65 (6.4)
Daily lifestyle, *n* (%)	769 (75.8)

^a^Proportions do not total to 100 % as respondents may chose more than one category

**Fig. 2:**
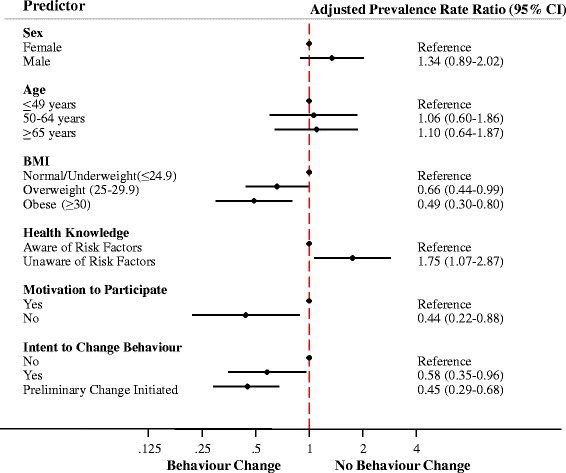
Adjusted prevalence rate ratio (PRR) for the association between participant characteristics and likelihood of behaviour change

We identified four significant predictors of behaviour change (Fig. 2). Individuals classified as overweight (BMI 25.0–29.9 kg/m^2^) and obese (BMI ≥ 30 kg/m^2^) were more likely to make a behaviour change (PRR 0.66, 95 % CI 0.44–0.99 and PRR 0.49, 95 % CI 0.30–0.80) as compared to those with a normal or underweight BMI (≤ 24.9 kg/m^2^). Further, participants unaware of the risk factors for CKD were less likely (PRR 1.75, 95 % CI 1.07–2.87) to make a behaviour change. Conversely, respondents who reported no particular motivation to participate in the screening events were more likely (PRR 0.44, 95 % CI 0.22–0.88) to make a behaviour change following the screening event. Finally, individuals who indicated intent to make health behaviour changes during the pre-screening survey were more likely to self-report making a behaviour change (PRR 0.58, 95 % CI 0.35–0.96) and those who said they had initiated preliminary behaviour changes were more likely to continue to make health behaviour changes (PRR 0.45, 95 % CI 0.29–0.68).

**Fig. 3:**
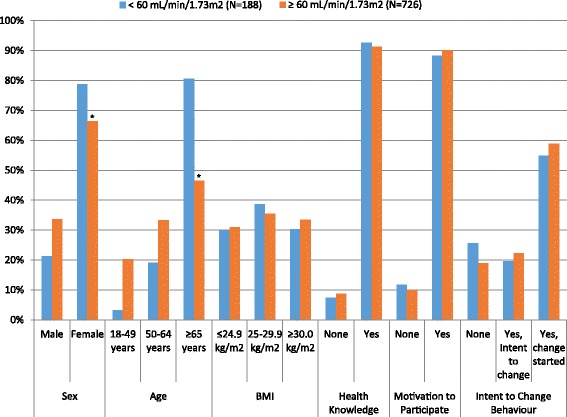
Proportion of participants who self-reported behaviour change by participant characteristics and eGFR category. *denotes statistically significant differences in proportions

Within our stratified analysis, the proportion of participants who self-reported a behaviour change was similar amongst those with eGFR < 60 and ≥ 60 mL/min/1.73 m^2^ for most patient characteristics (Fig. 3). However, a significantly higher proportion of females with eGFR < 60 mL/min/1.73 m^2^ (79 vs. 66 %, respectively (*p* < 0.05)) and individuals over 65 years old with eGFR < 60 mL/min/1.73 m^2^ (78 vs. 46 %, respectively (*p* < 0.05) reported self-reported behaviour change as compared to the participants with eGFR ≥ 60 mL/min/1.73 m^2^. Finally, results were similar in a sensitivity analysis excluding the 156 participants who self-reported having kidney problems and had an eGFR of <60 mL/min/1.73 m^2^.

## Discussion

In this national targeted screening programme to identify patients with unrecognized CKD, we found that individual counselling and goal setting may be an effective strategy to elicit a self-reported health behaviour change. We were also able to identify specific subgroups that could be targeted for this type of intervention. Specifically, participants unaware of the risk factors for CKD (limited health knowledge) were less likely to make a health behaviour change. However, individuals who were clinically overweight or obese, those with no self-identified motivation to participate in the screening event, and those who indicated an intent to change their behaviour were more likely to report a health behaviour change. Results were similar for patients with eGFRs of < 60 and ≥ 60 mL/min/1.73 m^2^ except for age and gender, where a higher proportion of women over 65 years of age with eGFR < 60 mL/min/1.73 m^2^ made a behaviour change as compared to their eGFR ≥ 60 mL/min/1.73 m^2^ counterparts.

Behaviour change interventions aim to promote healthy lifestyles and improve the uptake and optimal use of effective clinical services using a ‘coordinated set of activities designed to change specified behaviour patterns [17].’ Unfortunately, a combination of paucity of evidence, heterogeneous interventions, and poor reporting [28] leads to difficulty ascertaining the effectiveness of behaviour change interventions within CKD populations [25]. Although multifaceted educational interventions used to support behaviour change [21] have been shown to be effective in lowering blood pressure, improving blood sugars, and increasing health knowledge for various chronic conditions (diabetes and hypertension) [18], research to date has only shown effectiveness in improving knowledge [19] and prompting belief changes [20] within CKD. Given the difficulty in designing effective behaviour change interventions [17], recent evidence suggests that these interventions should be tailored to the individual and their disease trajectory [21, 29]. In fact, an individualised nutritional counselling intervention reported significant reductions in self-reported symptoms and problems associated with kidney disease in a pre-dialysis CKD population which shows promise for individual counselling in CKD [24]. Further research is required to understand the use of behaviour change interventions for patients with CKD. Our study highlights the use of individual counselling and goal setting to promote behaviour change following a targeted screening clinic.

Our results suggest that individual counselling and goal setting provided during targeted screening may be effective in eliciting behaviour change in certain groups of participants. We found that participants who were overweight or obese were more likely to change their behaviour, which could be attributed to a realization of poor health status at the screening event. In fact, recent evidence suggests goal setting is associated with weight reduction in patients with diabetes [30]. However, further research is required to determine if these interventions result in sustained long-term behaviour change.

Overall, the SeeKD individual counselling and goal setting intervention provided knowledge and skills on risk factors for kidney disease and prevention strategies (capability), external factors to prompt behaviour (opportunity), as well as some motivation (habitual or emotional processes to direct behaviour) to participants; all which differentially affected participant groups. For example, participants with an intent to change likely required opportunity and additional motivation, while those who had begun preliminary changes were simply reinforced to continue their behaviour change, thus highlighting pre-existing motivation in both groups. This is consistent with Proshaska and DiClemente’s model on the stages of behaviour change [31]. Participants with an intent to change are in the ‘preparation’ stage, while those who had begun preliminary changes would be in the beginning of the ‘action’ stage [31, 32].

Further, participants with no health knowledge of CKD (unaware of risk factors) were less likely to make a behaviour change. While this group may have low health literacy, which is associated with poor health outcomes and poor use of healthcare services [33], we cannot overlook the potential confounding effect of socioeconomic status [34]. Unfortunately, this information was not collected at the screening events. Finally, participants with no self-identified motivation to participate may be generally unaware of their personal health status but given the knowledge and skills, accompanied by externally derived motivation, are able to leverage the opportunity to make a behaviour change.

Our work suggests that future screening programmes may consider using individual counselling as a component of a health behaviour change intervention, but perhaps a different intervention is necessary when targeting individuals with low health knowledge. In general, counselling sessions should first identify the specific behaviour(s) for change and, using a behaviour framework [35], design an intervention focused on improving the uptake of knowledge and skills and simultaneously increasing motivation and empowerment [36] in order to improve the extent of behaviour change and engage those less likely to change. Given that many of the commitments to change fell within the ‘soft’ categories (e.g. dietary and lifestyle modifications), additional work is required to identify strategies aimed at more risky health behaviours such as smoking and alcohol consumption.

Though it is difficult to determine whether counselling sessions provided at a screening event would be similar to what is currently provided by physicians/nurses/pharmacists during clinic visits, it is likely that clinical discussions are more ad hoc and variable. Using a standardized tool that focuses on the needs of the patient may be promising but requires further research and evaluation before implementation in clinical practice. Further, the collection of additional participant information related to tolerability and satisfaction may also be important considerations in the adoption of such an intervention within a clinical setting.

Consideration should be given to limitations of the SeeKD screening programme when interpreting these results. As all participants screened for CKD were provided with this relatively short intervention (~20-min individual counselling session) and given the lack of a control group, we are unable to determine the true effectiveness of the behaviour change intervention. There may also be volunteer bias as participants self-selected to participate and may be systematically different from those who did not participate [37]. This is evident by the respondent characteristics, where the majority of participants were older females who participated due to a personal concern for their health. Follow-up bias is also of concern as survey respondents differed from the original study population. These potential selection biases may limit generalizability of the study population to the Canadian population at risk for CKD. Social desirability bias, a type of reporting bias whereby participants have a tendency to present a favourable image of themselves (e.g. overreport behaviour change) is of particular concern given the high proportion of participants who self-reported a behaviour change (89.8 %). However, this unrealistic positive response rate may also be driven by questionnaire design where participants did not explicitly have the option of stating no behaviour change.

## Conclusions

In this national survey of participants with risk factors for CKD, we found that the use of individual counselling and goal setting may be an effective intervention for stimulating behaviour change. This study highlights the importance of targeting specific groups with behaviour change interventions for optimal uptake. However, the current findings should be interpreted with caution given the study limitations. Despite the high rate of reported behaviour change amongst participants, future research is required to determine the key components of individual counselling as a behaviour change intervention, particularly within CKD populations.

## Abbreviations

BMI, body mass index; CI, confidence interval; CKD, chronic kidney disease; eGFR, estimated glomerular filtration rate; PRR, prevalence rate ratio; SD, standard deviation; SeeKD, See Kidney Disease targeted screening programme

## Appendix 1

### Behaviour change intervention

The behaviour change intervention, defined as an individual counselling and goal setting session conducted at each of the SeeKD screening events, followed the SeeKD protocol developed by the Kidney Foundation of Canada. The behaviour change intervention was carried out by a registered nurse, a pharmacist, or a dietician with experience in CKD, who tailored recommendations and strategies for behaviour change to each participant based on their risk factors and clinical measurements documented on their health data form. These sessions were allotted 20 min for discussion of kidney disease and how the participant may reduce their risk of developing CKD and CKD-related complications. Each screening event included a private counselling area. Brochures on kidney disease, its risk factors and prevention, generated by the Kidney Foundation of Canada, as well as copies of the Canada’s Food Guide and Canada Fitness Guide, were also provided to participants. These documents were offered in Chinese, Punjabi, French, and English in screening events across Canada. However, some regions (Ontario) were able to translate the brochures into the participants’ language of preference (i.e. Vietnamese, Korean, and Cambodian).

## Appendix 2

**Table 3:** Comparison of respondents to non-respondents

	Respondents (*N* = 1129^a^)	Non-respondents (*N* = 3265^a^)
Gender, male, *n* (%)	337 (29.9)	1119 (34.4)
Age (years), mean (SD)	63.8 (14.3)	56.5 (15.4)
CKD (eGFR < 60 mL/min/1.73 m^2^), *n* (%)	208 (20.6)	509 (17.3)
Motivation for participating, *n* (%)		
Concern for personal health status	618 (54.7)	1761 (53.9)
Influence from external source	227 (20.1)	803 (24.6)
Recruitment efforts	361 (32.0)	789 (24.2)
None	110 (9.7)	424 (13.0)
Self-reported risk factors, *n* (%)		
Diagnosed diabetes	274 (24.3)	693 (21.2)
Diagnosed hypertension	524 (45.5)	1186 (36.3)
Problems with kidneys	156 (13.8)	422 (12.9)
High-risk ethnic groups	509 (45.1)	2026 (62.1)
Vascular disease	268 (23.7)	614 (18.8)
Family history of kidney problems	166 (14.7)	454 (13.9)
Smoking or tobacco use	128 (11.3)	541 (16.6)
Knowledge of risk factors for CKD, *n* (%)		
Yes	1017 (90.1)	2862 (87.7)
No	110 (9.7)	403 (12.3)
Body mass index, *n* (%)		
Normal/underweight (≤ 24.9 kg/m^2^)	385 (34.1)	1015 (31.1)
Overweight (25.0–29.9 kg/m^2^)	376 (33.3)	1057 (32.4)
Obese (≥ 30.0 kg/m^2^)	315 (27.9)	1070 (32.8)

*SD* standard deviation, *CKD* chronic kidney disease

^a^Denominator varied for each variable depending on the number of participants with complete data available
